# A Genome-Wide Association Study Confirms *VKORC1*, *CYP2C9*, and *CYP4F2* as Principal Genetic Determinants of Warfarin Dose

**DOI:** 10.1371/journal.pgen.1000433

**Published:** 2009-03-20

**Authors:** Fumihiko Takeuchi, Ralph McGinnis, Stephane Bourgeois, Chris Barnes, Niclas Eriksson, Nicole Soranzo, Pamela Whittaker, Venkatesh Ranganath, Vasudev Kumanduri, William McLaren, Lennart Holm, Jonatan Lindh, Anders Rane, Mia Wadelius, Panos Deloukas

**Affiliations:** 1 Wellcome Trust Sanger Institute, Hinxton, United Kingdom; 2 Uppsala Clinical Research Centre, Uppsala, Sweden; 3 Department of Clinical Pharmacology, Karolinska Institute, Karolinska University Hospital, Stockholm, Sweden; 4 Department of Medical Sciences, Clinical Pharmacology, Uppsala University Hospital, Uppsala, Sweden; Queensland Institute of Medical Research, Australia

## Abstract

We report the first genome-wide association study (GWAS) whose sample size (1,053 Swedish subjects) is sufficiently powered to detect genome-wide significance (*p*<1.5×10^−7^) for polymorphisms that modestly alter therapeutic warfarin dose. The anticoagulant drug warfarin is widely prescribed for reducing the risk of stroke, thrombosis, pulmonary embolism, and coronary malfunction. However, Caucasians vary widely (20-fold) in the dose needed for therapeutic anticoagulation, and hence prescribed doses may be too low (risking serious illness) or too high (risking severe bleeding). Prior work established that ∼30% of the dose variance is explained by single nucleotide polymorphisms (SNPs) in the warfarin drug target *VKORC1* and another ∼12% by two non-synonymous SNPs (*2, *3) in the cytochrome P450 warfarin-metabolizing gene *CYP2C9*. We initially tested each of 325,997 GWAS SNPs for association with warfarin dose by univariate regression and found the strongest statistical signals (*p*<10^−78^) at SNPs clustering near *VKORC1* and the second lowest p-values (*p*<10^−31^) emanating from *CYP2C9*. No other SNPs approached genome-wide significance. To enhance detection of weaker effects, we conducted multiple regression adjusting for known influences on warfarin dose (*VKORC1*, *CYP2C9*, age, gender) and identified a single SNP (rs2108622) with genome-wide significance (*p* = 8.3×10^−10^) that alters protein coding of the *CYP4F2* gene. We confirmed this result in 588 additional Swedish patients (*p*<0.0029) and, during our investigation, a second group provided independent confirmation from a scan of warfarin-metabolizing genes. We also thoroughly investigated copy number variations, haplotypes, and imputed SNPs, but found no additional highly significant warfarin associations. We present power analysis of our GWAS that is generalizable to other studies, and conclude we had 80% power to detect genome-wide significance for common causative variants or markers explaining at least 1.5% of dose variance. These GWAS results provide further impetus for conducting large-scale trials assessing patient benefit from genotype-based forecasting of warfarin dose.

## Author Summary

Recently, geneticists have begun assaying hundreds of thousands of genetic markers covering the entire human genome to systematically search for and identify genes that cause disease. We have extended this “genome-wide association study” (GWAS) method by assaying ∼326,000 markers in 1,053 Swedish patients in order to identify genes that alter response to the anticoagulant drug warfarin. Warfarin is widely prescribed to reduce blood clotting in order to protect high-risk patients from stroke, thrombosis, and heart attack. But patients vary widely (20-fold) in the warfarin dose needed for proper blood thinning, which means that initial doses in some patients are too high (risking severe bleeding) or too low (risking serious illness). Our GWAS detected two genes (*VKORC1*, *CYP2C9*) already known to cause ∼40% of the variability in warfarin dose and discovered a new gene (*CYP4F2*) contributing 1%–2% of the variability. Since our GWAS searched the entire genome, additional genes having a major influence on warfarin dose might not exist or be found in the near-term. Hence, clinical trials assessing patient benefit from individualized dose forecasting based on a patient's genetic makeup at *VKORC1*, *CYP2C9* and possibly *CYP4F2* could provide state-of-the-art clinical benchmarks for warfarin use during the foreseeable future.

## Introduction

Warfarin is the most widely prescribed anticoagulant for reducing thromboembolic events that often give rise to stroke, deep vein thrombosis, pulmonary embolism or serious coronary malfunctions [1]. A combination of genetic and non-genetic factors cause Caucasians to exhibit 20-fold interindividual variation in required warfarin dose needed to achieve the usual therapeutic level of anticoagulation as measured by the prothrombin international normalized ratio or INR [2]–[4]. Thus, in the absence of information (genotypic, clinical, etc.) for predicting each patient's required warfarin dose, initial prescribed doses may be too low (risking thrombosis) or too high (risking over-anticoagulation and severe bleeding). Warfarin's risk of serious side effects, narrow therapeutic range, and wide interindividual variation in warfarin dose have focused attention on the need to better predict dose in the initial stage(s) of treatment.

We and others have shown that the warfarin drug target *VKORC1* (vitamin K epoxide reductase complex, subunit 1) contains common polymorphisms that account for a major portion (∼30%) of the variance in required warfarin dose [5],[6], and we have recently evaluated ∼1500 Swedish patients of the Warfarin Genetics (WARG) cohort in the largest study to date showing likely patient benefit from genetic forecasting of dose [3]. The study confirmed that SNPs in *VKORC1* and in the warfarin-metabolizing gene *CYP2C9* (cytochrome P450, family 2, subfamily C, polypeptide 9) predict ∼40% of dose variance while non-genetic factors (age, sex, etc.) jointly account for another ∼15%. The robust and now widely replicated associations of warfarin dose with *VKORC1* and *CYP2C9* have provided one of the most successful applications of pharmacogenetics to date [7] and offer promise for genetic predication of required dose in a clinical setting [3].

Knowledge of major predictors of warfarin dose also impacts the methodology for finding further dose-related genes. In early candidate gene work with a small sample of 201 patients [8], we noted that univariate regression (with tested SNP as the only dose predictor) could statistically detect warfarin association with *VKORC1* and with one of two non-synonymous *CYP2C9* SNPs (*3) known to influence warfarin dose (Table 1 in [8]). However, a second non-synonymous *CYP2C9* SNP (*2) with known but weaker influence on warfarin dose was not detected by univariate regression, but *2 was statistically significant in multivariate regression adjusted for the other known genetic and non-genetic predictors of dose (Table 3 in [8]). These empirical results in a small warfarin sample provided a signpost underscoring the potential importance of multivariate regression for detecting weak effects in studies now searching for additional warfarin genes across the genome.

**Table 1:** Association (*p*-value) of SNPs tested by univariate regression or multiple regression with progressive addition of known dose predictors^a^.

Predictors in regression analysis	Tested SNP				
	*VKORC1*	CYP2C9*3	CYP2C9*2	*CYP4F2*	Distribution of all SNPs
	rs9923231	rs1057910	rs1799853	rs2108622	
None	5.4E-78	4.5E-17	8.8E-13	1.6E-05	Figure 1A
Age, sex	7.3E-97	1.2E-24	2.4E-14	4.8E-06	–
Age, sex, *VKORC1*	–	3.8E-43	1.0E-15	4.6E-07	–
Age, sex, *VKORC1*, CYP2C9*3	–	–	1.4E-26	8.3E-08	–
Age, Sex, *VKORC1*, CYP2C9*3 and *2	–	–	–	8.3E-10	Figure 1B

a Linear regression on warfarin dose was calculated for the 1,053 GWAS subjects.

A genome-wide association study (GWAS) enables a systematic search of the entire genome for genetic factors that cause any inherited trait. This method has successfully identified susceptibility loci for common diseases [9], and is beginning to be applied to pharmacogenomics. A recent warfarin GWAS in 181 patients did not detect other genetic factors with major effects on warfarin dose beyond *VKORC1* [10] but was underpowered for identifying loci with a moderate contribution. We have now genotyped 325,997 SNPs in 1053 patients of the WARG cohort and here report the first GWAS that is sufficiently powered to detect additional genetic factors that may only modestly influence warfarin dose.

## Results

**Figure 1:**
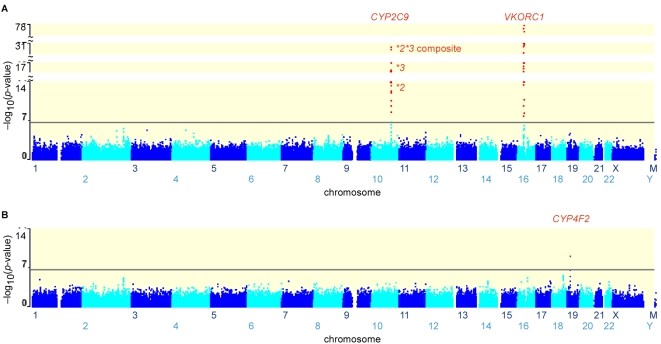
P-values for each GWAS SNP tested for association with warfarin dose. Horizontal axis shows SNP location and vertical axis is −log10(p-value) for each SNP tested by univariate regression (A) or multivariate regression (B). Red dots and red lettering show SNPs and implicated genes with p-values beyond the genome-wide significance threshold (1.5×10^−7^) which is denoted by a horizontal line. (A) Univariate regression shows genome-wide significant association to SNPs clustering near the warfarin drug target *VKORC1* (e.g., *P* = 5.4×10^−78^, rs9923231) and near the warfarin-metabolizing gene *CYP2C9* (*P* = 4.5×10^−17^ for non-synonymous *3 SNP rs1057910, *P* = 8.8×10^−13^ for non-synonymous *2 SNP rs1799853, *P* = 3.1×10^−31^ for *2*3 “composite” SNP rs4917639). (B) Multivariate regression adjusting for the contributions of *VKORC1* and *CYP2C9* had greater power than univariate regression and detected genome-wide significant association to the *CYP4F2* gene (*P* = 8.3×10^−10^, non-synonymous SNP rs2108622).

Figure 1A and the first line of Table 1 summarize results of testing 325,997 GWAS SNPs for association with warfarin dose by univariate regression. The strongest associations were at multiple SNPs in and near *VKORC1* (Figure 1A) with the lowest p-value given by rs9923231 (*P* = 5.4×10^−78^). In prior fine-mapping of the *VKORC1* locus [8], we identified rs9923231 as one of three SNPs located in introns or immediately flanking *VKORC1* that exhibit almost perfectly concordant genotypes yielding pairwise linkage disequilibrium (LD) *r* ^2^≈1 and which define the warfarin-sensitive A-T-T haplotype at rs9923231-rs9934438-rs2359612 (see also [11]). These highly concordant SNPs were the best predictors of warfarin dose in our previous study and in this GWAS analysis (p<5.4×10^−78^) and completely accounted for the dose variance explained by all other fine-mapping SNPs near *VKORC1* [8]. The group of SNPs with the second lowest univariate p-values clustered around *CYP2C9* which contains two non-synonymous exonic SNPs whose minor alleles (*2, *3) impair warfarin metabolism and are well known to be associated with warfarin dose. In our previous work [8], we discovered an unusual SNP (rs4917639) whose minor allele is almost perfectly associated with the “composite” *CYP2C9* allele formed by combining *2 and *3 into a single allele. Indeed, the GWAS results (1053 subjects) confirmed that LD is nearly perfect (pairwise *r* ^2^≈1.0) between rs4917639 and the composite of *2 and *3. Thus, the highly significant univariate result for rs4917639 (*R* ^2^ = 0.121, p<3.1×10^−31^) reflects the combined effect of CYP2C9*2 rs1799853 (*R* ^2^ = 0.038, p<8.8×10^−13^) and CYP2C9*3 rs1057910 (*R* ^2^ = 0.080, p<4.5×10^−17^). Figure 1A therefore indicates p-values for this composite SNP as well as for *2 and *3.

Figure 1B and Table 1 (lines 2 to 5) show the results of multivariate regression analysis in which individual SNPs were tested for association with warfarin dose after adjustment for established genetic and non-genetic predictors of dose. The only SNP reaching genome-wide significance (p<1.5×10^−7^) was a non-synonymous SNP (rs2108622) in exon 2 of *CYP4F2* (cytochrome P450, family 4, subfamily F, polypeptide 2) introducing a Val to Met amino acid change at position 433 (V433M). SNP rs2108622 predicts additional dose variance (∼1.1%) that is independent of the variance already explained by *VKORC1* and *CYP2C9*. As noted in the Introduction, our early studies with a small sample of 201 Swedish patients failed to detect the weak CYP2C9*2 effect on dose by univariate regression but *2 was significant in multiple regression [8]. The results in Table 1 with rs2108622 of *CYP4F2* show the same phenomenon with a p-value of 1.6×10^−5^ in univariate regression (line 1) but progressively lower p-values as known predictors are added to the multivariate model so that for the full model a p-value of 8.3×10^−10^ is achieved which is far below genome-wide significance (p<1.5×10^−7^). The *CYP4F2* association was further confirmed by testing an independent replication panel of 588 Swedish warfarin patients who gave a multivariate p-value of 0.0029 and a total overall p-value of 3.3×10^−10^ when combined with the GWAS subjects (Table 2). During preparation of this paper, a candidate gene study of drug-metabolizing and transporter genes independently discovered the association of rs2108622 and *CYP4F2* with warfarin dose, providing further confirmation [12].

**Table 2:** Multiple regression analysis of warfarin dose in the GWAS, replication and combined panels.

Predictor	WARG GWAS (1053)			Replication (588)			Combined (1641)		
	Effect on dose^b^	*R* ^2^	*P*-value	Effect on dose^b^	*R* ^2^	*P*-value	Effect on dose^b^	*R* ^2^	*P*-value
	Estimate (95% CI)			Estimate (95% CI)			Estimate (95% CI)		
*VKORC1* rs9923231 (C->T, 0.402)^a^	−0.96 (−1.03, −0.89)	0.283	1.6E-122	−0.99 (−1.09, −0.88)	0.284	5.0E-62	−0.97 (−1.02, −0.91)	0.283	2.7E-181
CYP2C9*3 rs1057910 (Ile359Leu, 0.070)^a^	−1.13 (−1.26, −1.00)	0.075	2.6E-55	−1.08 (−1.27, −0.89)	0.089	2.3E-26	−1.11 (−1.22, −1.00)	0.080	2.6E-79
CYP2C9*2 rs1799853 (Arg144Cys, 0.109)^a^	−0.63 (−0.74, −0.52)	0.048	1.7E-28	−0.40 (−0.55, −0.24)	0.023	5.5E-07	−0.54 (−0.63, −0.45)	0.038	1.1E-31
*CYP4F2* rs2108622 (Val433Met, 0.240)^a^	0.25 (0.17, 0.33)	0.016	8.3E-10	0.16 (0.05, 0.27)	0.005	c0.0029	0.21 (0.14, 0.27)	0.011	3.3E-10
Age	−0.04 (−0.04, −0.03)	0.170	1.9E-63	−0.03 (−0.04, −0.03)	0.129	1.7E-31	−0.04 (−0.04, −0.03)	0.155	1.2E-111
Sex (male)	0.35 (0.25, 0.45)	0.017	7.6E-12	0.25 (0.10, 0.40)	0.009	0.001	0.30 (0.22, 0.38)	0.013	1.6E-12

a In parenthesis are major/minor allele, and minor allele frequency.

b Effect of individual predictor on dose is indicated by regression coefficient and 95% confidence interval, proportion of explained variance (*R* ^2^) and *P*-value.

c Association in same direction as GWAS was assessed by a one-tailed test.

To increase the power of our multivariate regression model and possibly detect additional weak effects, we added *CYP4F2* (rs2108622) to the model as a predictor and conducted further analyses. First, we retested the GWAS SNPs, but no new SNPs reached genome-wide significance and there was also no apparent excess of SNPs at lower significance thresholds (Figure S1). We also tested warfarin association with haplotypes and with ungenotyped SNPs imputed at 2.2 million HapMap SNPs, but no haplotype or imputed SNP approached genome-wide significance in a genomic region not containing *VKORC1*, *CYP2C9* or *CYP4F2*. To explore whether copy number variations (CNVs) detectable by the HumanCNV370 array might influence warfarin dose, we used rigorous quality control and retained 879 samples calling 2530 CNVs (see Materials and Methods). None of the CNV loci were significantly associated with dose after correction for multiple testing (lowest CNV p-value was 1.1×10^−4^ which exceeds 0.05/2530≈2.0×10^−5^). We note that probe density in many of the detected CNVs is not optimal for conducting association analyses and these results should therefore be viewed as preliminary.

Finally, after excluding SNPs near *VKORC1*, *CYP2C9* and *CYP4F2*, we identified 40 other loci containing one or more GWAS SNPs with p-values below 2.0×10^−4^ and we genotyped 40 SNPs representing these loci in a follow-up sample of 588 Swedish warfarin patients. However none of the 40 loci replicated for association with warfarin dose, the lowest p-value being 0.04 which is not significant after correction for 40 tests (Table S1). Having not found evidence for any additional genetic modulators of dose, we examined the entire data set (GWAS plus followup samples) for evidence of statistical interaction between pairs of the established dose predictors (*VKORC1, CYP2C9, CYP4F2*, age, sex). None of the pairs exhibited statistically significant interaction after p-values were corrected for the 15 interaction tests (Table S2).

We also performed a GWAS for a secondary trait (“over-anticoagulation”) which we previously found was associated with *VKORC1* and *CYP2C9* in a candidate gene study [3]. By titrating warfarin dose, physicians attempt to achieve a target level of anticoagulation determined by a reading of 2.0 to 3.0 for the prothrombin international normalized ratio (INR), which is the ratio of time required for a patient's blood to coagulate relative to that of a reference sample. However over-anticoagulation (defined as an INR above 4.0) sometimes occurs and, using Cox regression, our GWAS tested for SNP association with the occurrence of over-anticoagulation in patients during the first 5 weeks of treatment (see Materials and Methods: Association testing of SNPs and haplotypes). We observed genome-wide significant association (p<1.5×10^−7^) at several SNPs in and around *VKORC1* including rs9923231 (P = 8.9×10^−9^), but no other SNPs achieved genome-wide significance including CYP2C9*3 (p<4.0×10^−5^), CYP2C9*2 (p = 0.93), or the “composite” *2*3 SNP rs4917639 (p<0.007) (Figure S2). However we note that our previous candidate gene study evaluated a larger sample set (1496 WARG subjects) which yielded genome-wide significant association with over-anticoagulation for both *VKORC1* rs9923231 (*P* = 5.7×10^−11^) and CYP2C9*3 (*P* = 1.5×10^−9^) [3]. To explore whether these SNPs might cause over-anticoagulation independent of altering the required (i.e., administered) warfarin dose, we added required dose to the Cox regression model as a predictor of over-anticoagulation, and found that both *VKORC1* and CYP2C9*3 have a significant effect independent of dose (*P*<0.05) (Table S3).

## Discussion

We conducted the first GWAS sufficiently powered to detect DNA variants with a modest influence on the warfarin dose needed to achieve therapeutic anticoagulation. In univariate analysis of GWAS SNPs (Figure 1A), we identified extremely strong association signals (p = 10^−78^ to 10^−13^) at SNPs in and near *VKORC1* and *CYP2C9*, two genes already known to explain ∼30% and ∼12% of warfarin dose variance, respectively. By applying multivariate regression adjusting for known genetic and non-genetic predictors of dose (Figure 1B), we also detected genome-wide significance of p<8.3×10^−10^ at *CYP4F2* (rs2108622) that accounted for approximately 1.5% of dose variance. The increased power of multivariate regression to detect this modest effect is nicely illustrated in Table 1 which shows a higher univariate p-value for *CYP4F2* (p<1.6×10^−5^) but progressively lower multivariate p-values as known predictors of dose are added to the regression model. We confirmed the *CYP4F2* association in a second large sample set and the association was also reported by another group [12] during preparation of our work, thus fully establishing the genuine effect of *CYP4F2* (see also [10] where *CYP4F2* explained ∼1% dose variance with nominal p<0.043 significance). Although multivariate regression has not been widely used to increase power in other GWAS analyses because known genetic variants usually explain little phenotypic variance, the potential for power increase is perhaps obvious if known predictors *do* explain substantial variance. Thus multiple regression has, for example, been previously advocated for linkage analyses of line crosses [13],[14].

To estimate the multivariate regression power of our GWAS (1053 subjects), we used Equation 1 (see Materials and Methods) to calculate power to detect SNPs explaining specific magnitudes of variance (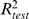) for warfarin dose (see Table 3). The table shows that power to achieve genome-wide significance (p<1.5×10^−7^) is essentially 100% for *VKORC1* rs9923231 (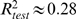), CYP2C9*3 (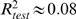) and CYP2C9*2 (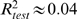), but power falls to ∼48% for *CYP4F2* rs2108622 (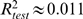). The table also shows that when *CYP4F2* is added to the multivariate model, a SNP accounting for 1.5% or 1.0% of the dose variance would have ∼82% or ∼41% power of being detected, respectively. Therefore we estimate that our GWAS had at least 80% power to detect warfarin-associated variants explaining at least 1.5% of the dose variance but 40% or less power to detect genome-wide significance if a variant accounts for 1% or less dose variance.

**Table 3:** Power to detect a dose-altering SNP as a function of its contribution to dose variance (*R* ^2^) and adjustment by other predictors in the multiple regression model^a^.

Predictors adjusted in multiple regression		Tested SNP					
		*VKORC1*	CYP2C9*3	CYP2C9*2	*CYP4F2*	Unknown	Unknown
description	total	rs9923231	rs1057910	rs1799853	rs2108622	SNP of	SNP of
	*R* ^2^	(*R* ^2^ = 0.283)	(*R* ^2^ = 0.080)	(*R* ^2^ = 0.038)	(*R* ^2^ = 0.011)	*R* ^2^ = 0.015	*R* ^2^ = 0.010
None	0.000	1.00	1.00	0.88	0.03	0.10	0.02
Age, sex	0.168	1.00	1.00	0.96	0.06	0.19	0.04
Age, sex, *VKORC1*	0.452	–	1.00	1.00	0.26	0.56	0.19
Age, sex, *VKORC1*, CYP2C9*3	0.532	–	–	1.00	0.40	0.73	0.31
Age, Sex, *VKORC1*, CYP2C9*3 and *2	0.570	–	–	–	0.48	0.81	0.39
Age, Sex, *VKORC1*, CYP2C9*3 and *2, *CYP4F2*	0.580	–	–	–	–	0.82	0.41

a Power calculations assumed a sample size of 1,053 subjects and significance level of 1.5E-7 as employed in our GWAS.

However it is important to emphasize that these power estimates assume that the dose-altering DNA variant is genotyped and tested directly or is indirectly detected through a marker in sufficiently high LD to the dose variant that the marker's 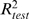 magnitude is detectable (Table 3). The assumption of directly testing the dose-altering variant is accurate for CYP2C9*2 and *3 which are each known to alter warfarin metabolism [15],[16] and is likely correct for *CYP4F2* rs2108622 which, like *2 and *3, changes protein coding sequence. However, to explore whether other dose-altering variants might be undetected due to insufficient LD with genotyped GWAS SNPs, we determined the relationship between the variance observed at a marker (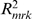) and at the causative variant (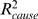) assuming pairwise LD of *r* ^2^ between the two polymorphisms (see Materials and Methods: How Much Does Linkage Disequilibrium Attenuate Association with a Quantitative Trait?). The relationship is given by Equation 3 in Materials and Methods (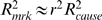) which is analogous to Pritchard and Prezworski's relationship (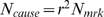) for the number of cases (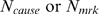) providing equal power in a case-control study that tests either the disease-causing SNP or a nearby marker [17]. To use the equation 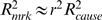 to estimate 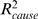 magnitudes for variants that might be *undetected* by our GWAS, we note that ∼90% of the GWAS SNPs had a minor allele frequency (MAF) above 10% in our warfarin subjects implying that a “rare” dose-altering variant (MAF≈1%–5%) would be covered at a likely maximum *r* ^2^ of only ∼0.1 to ∼0.5. This low *r* ^2^ coverage implies that rare variants could have 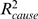 values (0.05 to 0.02) easily detected by regression testing of the variant itself, but unlikely to be detected through a GWAS marker since maximum 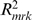 values could drop to 0.01 or much lower (see Equation 3 and Table 3). By contrast, “common” SNPs (MAF≥5%), which might also be dose variants, are covered by GWAS SNPs of this study at reasonably high *r* ^2^ values in most instances (*r* ^2^>0.8 or *r* ^2^>0.5 for ∼60% or ∼80% respectively of common SNPs [18] and *r* ^2^>0.9 for ∼90% of non-synonymous common SNPs [19] in HapMap Caucasians). We therefore conclude that our GWAS probably detected most common SNP variants explaining 1.5% or more of the warfarin dose variance, but may have failed to detect rarer variants that could individually explain up to 5% of dose variance. We further note that the HumanCNV370 array used in this study does not have the required marker complement to undertake a comprehensive GWAS of common CNVs.

As noted in the Introduction, the widely replicated warfarin dose associations with *VKORC1* and *CYP2C9* represent one of the most successful applications of pharmacogenetics to date. Our study together with that of Caldwell *et al*. [12] now also clearly demonstrates that *CYP4F2* (rs2108622) is a third gene that influences warfarin dose, but our GWAS and statistical analysis also implies that additional common SNP variants that influence dose may not exist in Caucasian populations. However, Caucasians might carry common variants with effects smaller than *CYP4F2* or rare variants whose effects are substantially larger than the ∼1% of dose variance explained by *CYP4F2*. Furthermore, other unidentified genes may influence warfarin dose in other ethnicities such as Asians or Africans, and some rare dose-altering variants in known genes such as *VKORC1* may exist in only a subset of populations of European descent [20]. Hence, future research could address ethnic differences in the genetic variants that influence warfarin dose as well as subtle intra-ethnic differences and admixture that may exist in European or other populations.

In a recent study [3], we highlighted the potential benefit of pre-treatment forecasting of required warfarin dose based on patient genotypes at *VKORC1* and *CYP2C9* together with non-genetic predictors of dose. Indeed, in August 2007, the US Food and Drug Administration (FDA) updated warfarin labeling to recommend initiating lower warfarin dose in some patients based on *VKORC1* and *CYP2C9* genotypes. However this recommendation is not a requirement due to a lack of large trials demonstrating warfarin patient benefit from dose forecasting (though two small trials [21],[22] do support such benefit; see also [23]–[27] for reviews and other trials). The results of our GWAS provide further impetus for conducting large-scale dose-forecasting trials by identifying *CYP4F2* as a third genetic predictor of dose and also by showing that additional major genetic predictors may not exist in Caucasians or may not emerge in the near-term. Hence, large-scale trials of patient benefit from dose forecasting based on *VKORC1* and *CYP2C9* (with possible inclusion of *CYP4F2* as a minor predictor) are likely to provide state-of-the-art clinical benchmarks for warfarin use during the foreseeable future.

## Materials and Methods

### Subjects and Clinical Data

The study subjects were 1053 Swedish patients collected for the WARG study [3] (http://www.druggene.org/). This is a multi-centre study of warfarin bleeding complications and response to warfarin treatment [28]. Anticoagulant response is measured by INR, which is the ratio of the time required for a patient's blood to coagulate relative to that of a reference sample. By titrating warfarin dose, physicians aim for a therapeutic INR reading between 2.0 and 3.0; thus the primary quantitative outcome for the GWAS was the mean warfarin dose (mg/week) given to a patient during a minimum series of three consecutive INR measurements between 2 and 3 [3]. As a secondary GWAS outcome, we also catalogued each patient for the occurrence or non-occurrence of “over-anticoagulation” during the first 5 weeks of treatment (defined as an INR reading above 4.0) and tested for genetic association which adjusted for the treatment day (1 to 35) of the over-anticoagulation event (see “Association testing” below). The clinical data collected by the WARG protocol included gender and age since each is a known non-genetic predictor of warfarin dose but did not include bodyweight and dietary information (e.g. vitamin K intake). Regression analysis of prescribed medication which can potentiate or inhibit warfarin action was not a statistically significant predictor of warfarin dose in the 1053 WARG GWAS subjects and hence was not included as a predictor variable in the multivariate regression analyses. The WARG study samples were previously described elsewhere [3],[4],[28],[29] as were the Uppsala followup samples [8]. The WARG and Uppsala studies received ethical approval from the Ethics Committee of the Karolinska Institute and the Research Ethics Committee at Uppsala University, respectively.

### Genotyping of SNPs and Sample Quality Control

From approximately 1500 WARG samples [3] examined for non-degradation and appropriate concentration of DNA (∼50 ng/µl), we randomly selected 1208 subjects for genotyping SNPs and CNV probes using the HumanCNV370 BeadChip array (Illumina). We excluded SNPs with MAF below 1%, call rate below 95%, or if call rate fell below 99% when MAF was below 5%. SNPs that departed from Hardy-Weinberg equilibrium (P<10^−6^) were also excluded. Subjects with genotyping call rate below 95% were also eliminated. Using iPLEX (Sequenom), subject identity (and associated phenotypic data) was cross-checked by genotyping four gender markers and 47 SNPs also carried on the HumanCNV370 array, enabling us to exclude ∼136 misidentified subjects. Sample quality (contamination) was further assessed by plotting each subject's genome-wide heterozygosity and eliminating outliers (with heterozygosity above or below the range of 0.312–0.372). After these quality control steps, a total of 1053 warfarin patients and 325,997 GWAS SNPs were retained for analysis. The GWAS SNPs included two SNPs not on the HumanCNV370 array but which are highly predictive of warfarin dose [rs9923231 (*VKORC1*) and rs1799853 (CYP2C9*2)] which we genotyped by TaqMan assay (Applied Biosystems).

### Defining CNV Regions

Although we retained 325,997 GWAS SNPs for association testing of SNPs, it should be noted that all ∼370,000 probes on the Human CNV370 array were used to define CNVs. Log R ratio values of probes were output from the BeadStudio software [30]. A loess correction was applied to each sample to remove local correlations or genomic wave [31]. The resultant genomic copy number profiles were then segmented using Circular Binary Segmentation [32]. Some samples displayed abnormally high numbers of segments indicating problems in DNA quantity or quality or hybridization. Samples were removed until the number of segments across all samples was approximately normal. Using this technique, 143 (14%) of samples were flagged as problematic. These samples were excluded when CNV regions were defined but included for association testing. Putative CNV were defined from segments by applying a threshold on the segment log R ratio. This threshold was asymmetric allowing for a differing response for deletions and duplications. The central peak of the segment log R ratio distribution was fitted and the threshold values obtained by taking values at ±5 standard deviations from the centre.

In order to define regions for association testing, merging of CNV across samples was performed. This was achieved by merging two putative CNV into a region if there was greater than 40% reciprocal overlap. This procedure defined 2530 CNV regions in total. Of these, most were singletons (54%) or low frequency, <3% (93%), while 820 (70%) of the non-singleton regions overlapped CNVs from the Database of Genomic Variants [33]. We tested all 2530 CNVs for association, because a CNV discovered as a “singleton” might well include multiple copies of a rare CNV allele in the study samples.

### Association Testing of SNPs and Haplotypes

At each SNP, genotypes were coded 0, 1 or 2 and the SNP was tested for association with the square root of warfarin dose [8] by either univariate or multivariate linear regression analysis conducted in PLINK [34] (http://pngu.mgh.harvard.edu/~purcell/plink/) or in R software (http://www.r-project.org/). We used the same regression analysis to test association with all HapMap SNPs not on the HumanCNV370 array by imputing ∼2.2 million SNPs using Beagle software [35] trained from genotypes of the 60 HapMap CEU parents [36]. We excluded SNPs whose imputed MAF was below 5% or differed by more than 5% with MAF of the CEU parents.

We also tested haplotypes for association with warfarin dose by two approaches: (1) each subject's warfarin dose residual (difference between observed and predicted dose based on the full multivariate regression model containing *CYP4F2*) was considered a quantitative trait value and tested for association with haplotypes defined across the genome in sliding windows of 2, 3 or 4 consecutive SNPs as implemented by PLINK software; (2) by scanning GWAS genotypes, Beagle software groups genetically related haplotypes into clusters which it then resolves into diallelic (SNP-like) “pseudo-markers” optimized for detecting phenotypic association. To test haplotypes, we evaluated the pseudo-marker genotypes of warfarin patients at 1.97 million pseudo-markers covering the genome by testing each pseudo-marker in the same multivariate regression framework used to test individual SNPs (as described in the preceding paragraph).

We tested for statistical interaction in modulating warfarin dose for each pair of established dose predictors (*VKORC1* rs9923231, CYP2C9*2 and *3, *CYP4F2* rs2108622, Age, Sex) using multivariate regression and R software as described above. An interaction term formed by multiplying the pair of predictor variables was added to the multivariate regression equation which contained only main effects of the 6 predictors, and standard ANOVA compared this main-effect model with the enhanced interaction model by testing for a statistically significant increase in explained dose variance. Interaction test p-values were considered statistically significant if below the Bonferroni cutpoint determined by correcting for the 15 interaction tests (i.e. p<0.0033≈0.05/15).

To test for association with over-anticoagulation (INR>4.0) during treatment days 1–35, we performed Cox proportional hazard regression on survival time (day of over-anticoagulation) using the survival library of R software. The GWAS data set of 1053 WARG subjects contained 215 subjects whose INR exceeded 4.0 during days 1–35 while the entire dataset of 1489 WARG subjects contained 312 such subjects.

### Association Testing of CNVs

For each CNV locus, association was tested with square root of warfarin dose by multivariate regression analysis in which subject copy number intensity was the CNV predictor of dose. This analysis differs from association testing with SNP genotypes since the two CNV alleles on homologous chromosomes generate one copy number intensity rather than a separate allele for each chromosome. As a QC strategy, we determined each subject's rank in the dataset for copy number intensity at each CNV on chromosome 17. This enabled us to differentiate the majority of subjects (whose individual distribution of ranks were approximately random and uniform) from 174 obvious outliers due to poor quality DNA (whose ranking distributions were “U-shaped” since their intensities strongly clustered at both high and low ranks). These 174 subjects were excluded from the primary CNV association analysis (with further confirmation of lower quality DNA for these subjects being their rough correspondence to the subjects with lower (<99%) SNP call rates). However, we also crosschecked the primary CNV analysis by conducting association testing on the dataset without excluding the 174 subjects and found no statistically significant association with warfarin dose at any CNV whether the dataset excluded or included the subjects. Association testing of the CNVs was executed using R software [37].

### Replication of *CYP4F2*

For the replication of *CYP4F2* rs2108622, we genotyped a panel of 588 warfarin patients consisting of 410 subjects from the WARG cohort [3] and 178 from the Uppsala cohort [38]. Table 2 shows regression on this pooled sample of 588 subjects. Separate results for each of the two panels are given in Table S4.

### Follow-Up of Moderately Significant SNPs

To possibly identify SNPs with genuine but weak associations to warfarin dose, we excluded *VKORC1*, *CYP2C9*, *CYP4F2* and identified 40 other GWAS loci for follow-up genotyping exhibiting multivariate regression p-values below 0.0002, and selected 40 SNPs representing these loci for genotyping. Only genotyped (not imputed) SNPs were chosen for follow-up. We genotyped the same 558 patients as in the *CYP4F2* replication using the iPLEX MassARRAY.

### Power Calculation

Suppose multiple regression analysis is conducted in *N* total samples by testing a SNP with coefficient of determination (i.e., explained variance) *R* ^2^ *_test_* after adjustment for known predictors whose total of coefficient of determination is *R* ^2^ *_knw_*. The probability (power) to detect the tested SNP at a significance level α equals:
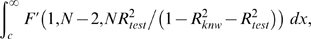
(1)where *F′*(1, *N*–2, *θ* ^2^) is the probability density function for an *F* distribution with 1, *N*-2 degrees of freedom and non-centrality parameter *θ* ^2^ (Section 28.28 in [39], Example 8.4 in [40]). Here the constant *c* satisfies the equation:
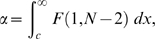
(2)where α is the significance level, and *F*(1, *N*–2) is the probability density function for a *F*-distribution of degree of freedom one and *N*–2.

### How Much Does Linkage Disequilbrium (LD) Attenuate Association with a Quantitative Trait?

Association with a quantitative trait (QT) becomes weaker for a marker SNP in LD with a SNP that alters the QT, and hence the association becomes more difficult to detect at the marker than at the QT-altering SNP. Here we quantify the LD attenuation for a QT when testing for association by linear regression (which includes the Cochran-Armitage trend test for dichotomous traits), and we obtain a result analogous to the LD attenuation for the Pearson Chi-square test for allelic association to dichotomous traits as in cases and controls [17]. If a causative QT-altering SNP has a coefficient of determination (i.e., explained variance) 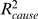 and is in pairwise LD of *r* ^2^ with a marker SNP, then the coefficient of determination for the marker SNP (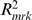) is approximated by:
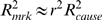
(3)


In other words, when testing a marker, the proportion of explained variance decreases by a factor of *r* ^2^.

To begin the proof of Equation 3, let the QT be represented by the random variable “*q*”, and let “*m*” and “*x*” be SNP genotypes (coded 0, 1, or 2) representing the marker and causative (QT-altering) SNP, respectively. The coefficients of determination 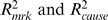 are equal to the square of two correlation coefficients (denoted by “Corr”) measuring the correlation of *m* or *x* with *q*:
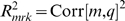
(4)

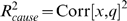
(5)


Also note that correlation between *genotypes* at the marker and causative SNP is given by another correlation coefficient:
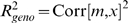
(6)


It is well known that the *partial* correlation coefficient of *m* and *q* conditioned on *x* is (equation 16.20, p. 649 in [41]):
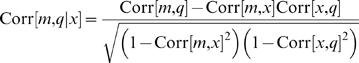
(7)


However, conditional on genotype at the causative SNP, marker *m* and the QT *q* would be uncorrelated (assuming *m* is not in LD with a second causative polymorphism) and thus the numerator of Equation 7 would be zero implying that:
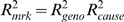
(8)


Based on prior work [42]–[44], we show in Text S1 that the squares of the genotypic correlation coefficient 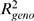 and LD correlation coefficient *r* ^2^ are approximately equal if the population is in Hardy-Weinberg equilibrium. Therefore, substituting *r* ^2^ for 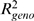 in Equation 8 gives Equation 3.

## Supporting Information

Figure S1 QQ plot for association of each GWAS SNP with warfarin dose. SNPs were tested for association with warfarin by regression analysis that adjusted for age, sex, and genotype at *VKORC1*, CYP2C9*2 and *3, *CYP4F2*. The QQ plot omits SNPs in loci already known to be associated with warfarin dose (*VKORC1*, *CYP2C9*, *CYP4F2*). The excess of SNPs with small p-values is minor: whereas 65.4 SNPs with *p*<0.0002 are expected, 70 were observed (1.069 times inflated).
(0.20 MB PDF)

Figure S2 Manhattan plot of GWAS results of testing for association with warfarin-induced over-anticoagulation. Horizontal axis is the genomic position, and vertical axis is minus log of p-value. Red dots above the gray line indicate association of genome-wide significance (*p*<1.5×10^−7^) at SNPs in the *VKORC1* locus such as rs9923231 (*p* = 8.9×10^−9^). However, no other loci achieved genome-wide significance. See main text for more details.
(0.07 MB PDF)

Table S1 Multivariate regression results for 40 SNPs followed-up after GWAS. Unlike rs2108622 of *CYP4F2*, none of these 40 SNPs exhibited statistical significance after correction for multiple testing. See main text for more details.
(0.03 MB XLS)

Table S2 Testing for statistical interaction between predictors of warfarin dose. After correcting for the 15 interaction tests, no pair of predictors exhibited statistically significant interaction. Data is for the combined panel of subjects (N = 1641). See main text for more details.
(0.02 MB XLS)

Table S3 Survival time analysis for incidence of over-anticoagulation within the first 5 weeks of treatment in the whole WARG cohort (N = 1489). See main text for more details.
(0.02 MB XLS)

Table S4 Multiple regression analysis of warfarin dose in WARG replication samples, WARG GWAS plus replication, or Uppsala replication samples. This table shows the same results displayed in Table 2 of the main text except that WARG and Uppsala subjects are separated into different subsets.
(0.02 MB XLS)

Text S1 For populations in Hardy-Weinberg Equilibrium, Linkage Disequilibrium r^2^ and Genotypic R^2^ are approximately equal.
(0.03 MB DOC)
