# Supplementary material for: A Genome-Wide Association Study Confirms VKORC1, CYP2C9, and CYP4F2 as Principal Genetic Determinants of Warfarin Dose
Source: PLoS Genet. 2009 Mar 20;5(3):e1000433. doi: 10.1371/journal.pgen.1000433 (PMC2652833; doi:10.1371/journal.pgen.1000433)

Supplementary Figure 1

A quantile-quantile plot for association of each SNP with warfarin dose adjusted for age, sex and genotypes for *VKORC1*, *CYP2C9*2*, **3* and *CYP4F2*. We omitted SNPs from the loci of *VKORC1* (chrm16, 30.4–31.5Mbp), *CYP2C9* (chrm10, 96.2–97.2Mbp) and *CYP4F2* (chrm19, 15.3–16.3Mbp). The excess of small *p*-value SNPs is subtle: whereas 65.4 SNPs with $p<0.0002$ are expected, 70 were observed (1.069 times inflated).

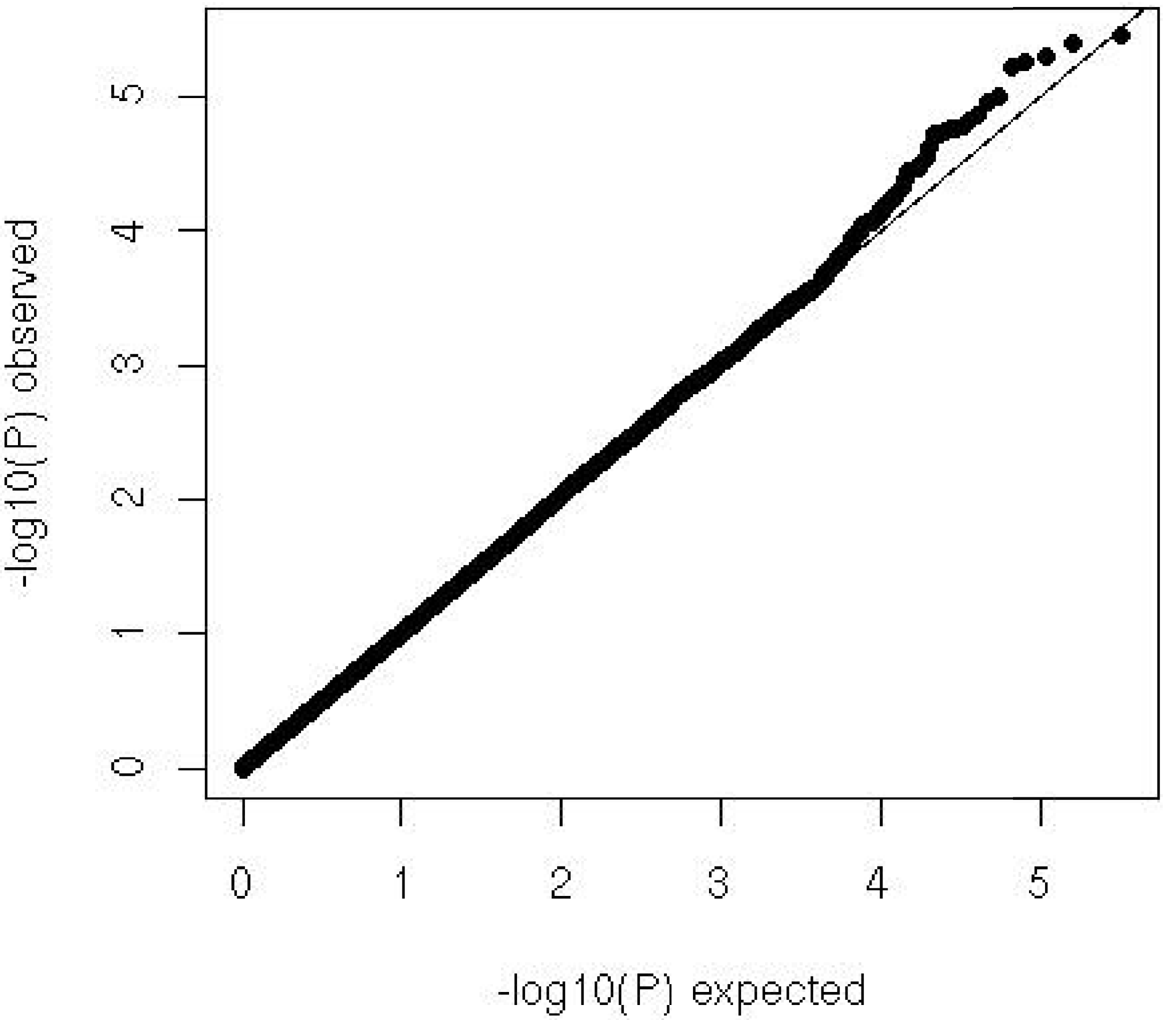

Supplement: Figure S1 — QQ plot for association of each GWAS SNP with warfarin dose. SNPs were tested for association with warfarin by regression analysis that adjusted for age, sex, and genotype at VKORC1, CYP2C9*2 and *3, CYP4F2. The QQ plot omits SNPs in loci already known to be associated with warfarin dose (VKORC1, CYP2C9, CYP4F2). The excess of SNPs with small p-values is minor: whereas 65.4 SNPs with p<0.0002 are expected, 70 were observed (1.069 times inflated). (0.20 MB PDF) [file pgen.1000433.s001.pdf]
