# Supplementary material for: A Genome-Wide Association Study Confirms VKORC1, CYP2C9, and CYP4F2 as Principal Genetic Determinants of Warfarin Dose
Source: PLoS Genet. 2009 Mar 20;5(3):e1000433. doi: 10.1371/journal.pgen.1000433 (PMC2652833; doi:10.1371/journal.pgen.1000433)

Supplementary Figure 2

Graphical summary of a GWA scan for incidence of over-anticoagulation. Horizontal axis is the genomic position, and vertical axis is minus the common logarithm of *p*-value. Red dots above the gray line indicate association of genome-wide significance ($p<1.5\times10^{-7}$). SNPs in the *VKORC1* locus, such as rs9923231 ($p=8.9\times10^{-9}$), attained genome-wide significance, but there were no significant association in other loci.

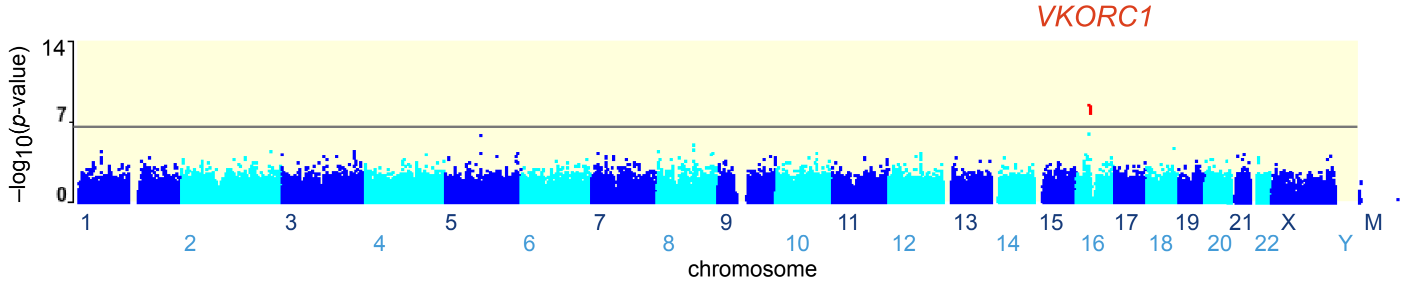

Supplement: Figure S2 — Manhattan plot of GWAS results of testing for association with warfarin-induced over-anticoagulation. Horizontal axis is the genomic position, and vertical axis is minus log of p-value. Red dots above the gray line indicate association of genome-wide significance (p<1.5×10^−7^) at SNPs in the VKORC1 locus such as rs9923231 (p = 8.9×10^−9^). However, no other loci achieved genome-wide significance. See main text for more details. (0.07 MB PDF) [file pgen.1000433.s002.pdf]
