# Supplementary material for: A Genome-Wide Association Study Confirms VKORC1, CYP2C9, and CYP4F2 as Principal Genetic Determinants of Warfarin Dose
Source: PLoS Genet. 2009 Mar 20;5(3):e1000433. doi: 10.1371/journal.pgen.1000433 (PMC2652833; doi:10.1371/journal.pgen.1000433)
Supplement: Text S1 — For populations in Hardy-Weinberg Equilibrium, Linkage Disequilibrium r2 and Genotypic R2 are approximately equal. (0.03 MB DOC) [file pgen.1000433.s007.doc]

## Text S1

Let “A” and “B” be alleles at two bi-allelic polymorphisms (e.g. SNPs) located close enough to be in LD. The standard LD coefficient (D_AB_) is transformed to the LD correlation coefficient (r) by the well known formula $\text{r}=\text{D}_{\text{AB}}/\sqrt{\text{P}_{\text{A}}(1-\text{P}_{\text{A}})\text{P}_{\text{B}}(1-\text{P}_{\text{B}})}$ where P_A_ and P_B_ are allele frequencies of A and B [42]. Similarly, Weir [42,43] described the “composite” disequilibrium coefficient (Δ_AB_) which he explained is similarly transformed to an analogous correlation coefficient (r*) by the formula $\text{r*}=\Delta_{\text{AB}}/\sqrt{\text{[P}_{\text{A}}(1-\text{P}_{\text{A}})+\text{D}_{\text{A}}\text{] [P}_{\text{B}}(1-\text{P}_{\text{B}})+\text{D}_{\text{B}}]}$ where D_A_ and D_B_ are Hardy-Weinberg disequilibrium coefficients. Whether the formula variables are for the entire population or a particular sample, Zaykin [44] and Weir [43] each noted that r* is the same as the formula for the (unsquared) correlation between genotypes ($\text{R}_{\text{geno}}$) at two loci; and thus$\text{R}_{\text{geno}}^{2}=(r*)^{2}$.

For a population in Hardy-Weinberg equilibrium (HWE), the population values of the four disequilibrium coefficients are such that Δ_AB_=D_AB_ and D_A_=D_B_=0; and Weir [43] also noted that the population values of r and r* become identical implying that $\text{(r*)}^{2}=\text{R}_{\text{geno}}^{2}=r^{2}$. Weir [43] also stated that the sample statistics of r and r* are normally distributed after Fisher’s z-transformation such that their sample means are equal under HWE while their sampling variances are 1/(2N-3) and 1/(N-3), respectively (where N is number of subjects in the sample). We therefore conclude that $\text{R}_{\text{geno}}^{2}\approx r^{2}$ for samples from populations in HWE for which sample size is sufficiently large (N>100) for accurate normal distribution under Fisher z-transformation with the approximation being very good for sample sizes as large as our GWAS (N>1000).
